# The Role of Diffusion-Weighted Magnetic Resonance Imaging in Differentiating Benign From Malignant Thyroid Nodules: A Descriptive Observational Study

**DOI:** 10.7759/cureus.30493

**Published:** 2022-10-19

**Authors:** Vijaykumar Monisha, N Rache Gowda, Sakalecha Anil Kumar

**Affiliations:** 1 Radiodiagnosis, Sri Devaraj Urs Medical College, Kolar, IND; 2 Radiodiagnosis, MVJ Medical College and Research Hospital, Bengaluru, IND

**Keywords:** apparent diffusion coefficient (adc), malignancy, thyroid cancer, magnetic resonance imaging, neoplasms

## Abstract

Background

Currently, no effective and reproducible imaging method can quantitatively differentiate between benign and malignant thyroid nodules. This study aimed to assess the role of diffusion-weighted imaging (DWI) in differentiating benign from malignant thyroid nodules.

Methodology

We conducted an observational study in the Department of Radiodiagnosis at R. L. Jalappa Hospital and Research Center, Kolar, India. The study was conducted from January 2020 to June 2021, among 43 patients with thyroid swelling or nodules. We recorded patient baseline data, pertinent clinical history, and relevant laboratory investigations. Patients underwent diffusion-weighted MRI of the neck. We used receiver operator curve analysis to determine the utility of the apparent diffusion coefficient (ADC) of the thyroid nodule in predicting the histopathological diagnosis. Data were analyzed using coGuide (V1.0.3, BDSS Corp., India).

Results

A total of 43 patients were enrolled in the study (35 women, 81.4%; 8 men, 18.6%). The mean (SD) age was 49.8 (14.3) years. Nodular hyperplasia was the most common histopathologic diagnosis of the nodule (25.5%), followed by lymphocytic thyroiditis (16.2%). The MRI and histopathological findings matched: both found 35 (81.4%) nodules as benign and 8 (18.6%) as malignant. The ADC value of thyroid nodules had excellent predictive validity in predicting malignancy, as indicated by the area under the curve of 0.925 (95% confidence interval [CI] 0.804-1.000; *p *< 0.001). The sensitivity was 87.50% (95% CI 47.35%-99.68%), specificity was 94.29% (95% CI 80.84%-99.30%), and the total diagnostic accuracy obtained was 93.02% (95% CI 80.94%-98.54%).

Conclusions

DWI with ADC measurement has the potential to differentiate between benign and malignant thyroid nodules.

## Introduction

In 2018, thyroid cancer (TC) was considered one of the common endocrine malignancies, constituting 3.3% of all neoplasms [1]. Since the past decade, incidence rates of TC have been increasing worldwide in high- and middle-income countries [2]. In India, TC incidence rates are estimated to be below the world average (5.4/100,000 in India vs. 6.7/100,000 worldwide) [3]. With early detection, follow-up, and active treatment, the 10-year survival rate can be up to 90% [4].

Differentiating benign from malignant thyroid nodules using noninvasive techniques is still a major problem despite the availability of diagnostic techniques such as ultrasonography and computed tomography scans of the neck. Neck ultrasonography can provide information about the size, shape, and composition of the nodule; margins; and presence of internal echogenic foci based on which Thyroid Imaging Reporting and Data Systems (TIRADS) scoring is given to the nodule, but still, there are no reliable criteria for distinguishing benign from malignant thyroid nodules. In addition, it is difficult to diagnose malignancy of the nodule when it is large or multinodular. Despite great improvement in diagnostic techniques such as thyroid ultrasound scan and CT scan of the neck, there is still a large problem in using a noninvasive and reliable technique to differentiate benign from malignant thyroid nodules. Recent developments in MRI techniques may be of diagnostic value in this regard. Diffusion-weighted imaging (DWI) is an emerging technique for the assessment of tumors, and the apparent diffusion coefficient (ADC) can be used to review tumor microstructure [5].

In imaging, diffusion is the random microscopic movement of water molecules, which is used as a sensitive parameter for tissue characterization at a microscopic level. Today, in vivo measurement of diffusion is possible with DWI and by calculation of ADC measurements. As MRI is sensitive to the diffusion of water protons in biological tissues, new technological developments can help obtain DWI. Intracellular and extracellular water balance is important for diagnosing stroke and for patient monitoring after stroke. Due to recent improvements in echo planar imaging techniques, DWI can now be used successfully in all areas with lesser probability of artifacts [6].

DWI has the potential to differentiate benign from malignant thyroid nodules. Meyer et al. conducted a systematic review and meta-analysis that found that DWI and ADC are valuable tools in distinguishing malignant and benign thyroid tumors [7]. They suggested using DWI as a routine presurgical diagnostic tool [7]. Chen et al. found that DWI was a noninvasive and accurate method for distinguishing between malignant and benign thyroid nodules [8]. Another meta-analysis suggested that DWI and ADC could be used as diagnostic tools in distinguishing malignant and benign thyroid nodules [9].

Literature on ADC as a quantitative parameter for distinguishing malignant tumors from benign thyroid nodules is limited in low-income countries such as India. Therefore, this study aimed to assess the role of DWI in differentiating benign from malignant thyroid nodules using ADC values and histopathology evaluation of thyroid nodules.

## Materials and methods

We conducted a hospital-based observational study among eligible patients who would have undergone an MRI scan of the neck in the Department of Radiodiagnosis at R.L. Jalappa Hospital and Research Center attached to Sri Devaraj Urs Medical College (SDUMC), Tamaka, Kolar, India. The study was conducted from January 2020 to June 2021. The sample size was estimated by using the proportion of patients with benign and malignant thyroid nodules detected by DWI from the study by Aghaghazvini et al. [10] using the formula Z_1_ − α/2 = 1.96 at 5% error alpha. As in most studies, p-values are considered significant below 0.05; therefore, 1.96 is used in the formula. P represents the expected proportion in the population based on previous studies or pilot studies, and d represents the absolute error or precision (p = 0.87; 1 − p = 0.13; d = 10%). Assuming a sensitivity of 87% with absolute precision of 10% confidence interval (CI) of 95%, the minimum sample size obtained was 43. All eligible subjects were sequentially recruited into the study using convenience sampling until the sample size was met.

The study included patients with thyroid swelling/nodule on clinical examination and patients with thyroid swelling/nodule on ultrasonographic examination. The study excluded patients in whom fine needle aspiration or previous instrumentation or biopsy was performed within the last three weeks. The institutional human ethics committee of the concerned institution approved the study (SDUMC/KLR/IEC/134/2019-20, October 11, 2019). Informed written consent was obtained from all the study participants; the risks and benefits involved in the study were explained to the participants before obtaining consent. The confidentiality of the study participants was maintained.

Baseline data were collected from the patients, and pertinent clinical history and relevant laboratory investigations were recorded. Our primary outcomes were MRI findings and the predictive validity of ADC in differentiating malignant and benign thyroid nodules. Our secondary outcomes were pathological findings that were obtained either after performing fine needle aspiration or after biopsy.

MRI of the neck was performed using a 1.5 T, 18-Channel MR Scanner (Magnetom Avanto, Siemens, Munich, Germany). We obtained axial turbo spin-echo T2-weighted (time of repetition [TR]/time of echo [TE], 2,500/75; matrix size, 307 × 384; field of view [FOV], 220 mm; and slice thickness, 4 mm, with a 1 mm intersection gap), coronal turbo spin-echo T2-weighted (TR/TE, 3,000/69; matrix size, 307 × 384; FOV, 240 mm; and slice thickness, 5 mm, with a 1 mm intersection gap), and axial T1-weighted (TR/TE, 746/11; matrix size, 307 × 384; FOV, 220 mm; and slice thickness, 4 mm, with a 1 mm intersection gap) images. DWI was obtained in the axial plane by single-shot spin-echo and echo-planar imaging using *b* values of 400 and 800 s/mm^2^ (TE, 70; TR, 5,400; slice thickness, 4 mm, with no intersection gap and signal acquisition number). After acquiring images, we drew regions of interest in every suspected nodule on ADC maps based on the size of the nodule, excluding the areas of calcification and necrosis, and cystic components.

Statistical analyses

We used mean and SD for quantitative variables and frequency and proportion for categorical variables. Categorical outcomes were compared between study groups using the Chi-squared and Fisher's exact tests. The utility of the thyroid nodule's ADC in predicting the histopathological diagnosis of the nodule was assessed by receiver operative curve (ROC) analysis. The area under the ROC curve and its 95% CI and *p*-value are presented. Based on the ROC analysis, 1.0 × 10^−3^ mm^2^/s was considered the cutoff value. The specificity, sensitivity, diagnostic accuracy, and predictive values with the decided cutoff value and their 95% CI are presented. We considered *p *< 0.05 as statistically significant. Data were analyzed using coGuide software (V.1.0.3; BDSS Corp., India) [11].

## Results

A total of 43 subjects were included in the final analysis. The mean (SD) age was 49.8 (14.3) years (range, 24-78 years); 8 (18.60%) patients were male, and 35 (81.40%) were female. Bilateral lobe involvement was observed in 13 (30.2%) patients. Multiple nodules were noted in 14 (32.5%) patients. A round nodule was noted in 25 (58.1%) patients, and an oval nodule was found in 12 (27.9%) patients. Most nodules (*n *= 31, 81.4%) had smooth margins. The proportion of solid and solid-cystic composition was 53.4% and 20.9%, respectively. (TIRADS or American Thyroid Association [ATA] thyroid classification is mainly used for USG classification of thyroid nodules. Here, in our case, we have performed an MRI for the assessment of thyroid nodules; hence, TIRADS classification was not considered.) Only 1 (2.3%) patient had a retrosternal extension of a very large thyroid nodule. We found calcifications in 11.6% and lymph nodes in 20.9% of the nodules (Table 1).

**Table 1:** Descriptive analysis of baseline parameters in the study population (N = 43).

Parameters	*n* (%)
Side (lobe) of involvement	
Left	16 (37.2)
Right	13 (30.2)
Both	13 (30.2)
Isthmus	1 (2.3)
Number of nodules	
One	24 (55.8)
Two	5 (11.6)
Multiple	14 (32.5)
Shape	
Round	25 (58.1)
Oval	12 (27.9)
Irregular	6 (13.9)
Margins	
Smooth	35 (81.4)
Irregular	6 (13.9)
Lobulated	2 (4.6)
Composition of the lesion	
Predominantly cystic	3 (6.9)
Predominantly solid	8 (18.6)
Solid cystic	9 (20.9)
Solid	23 (53.4)
Extrathyroidal extension	
Retrosternal extension	1 (2.3)
Calcifications	5 (11.6)
Lymph nodes	9 (20.9)

A total of 9 (20.9%) thyroid nodules showed restricted diffusion, and 34 (79%) did not show restricted diffusion on DWI with ADC. The mean value of ADC was 1.42 ± 0.45 × 10^−3^ mm^2^/s (range, 0.51 to 2.90 × 10^−3^ mm^2^/s). Nodular hyperplasia was reported in 25.5% of patients, lymphocytic thyroiditis in 16.2%, and the colloid nodule in 13.9% (*n *= 6). Table 2 presents other histopathology findings.

**Table 2:** Descriptive analysis of outcome parameters in the study population (N = 43). DWI, diffusion-weighted imaging; ADC, apparent diffusion coefficient

Parameters	*n* (%)
DWI	
Restricting	9 (20.9)
Nonrestricting	34 (79)
Pathological findings	
Nodular hyperplasia	11 (25.5)
Lymphocytic thyroiditis	7 (16.2)
Colloid nodule	6 (13.9)
Hashimoto's thyroiditis	5 (11.6)
Benign follicular lesion	4 (9.3)
Papillary thyroid carcinoma	4 (9.3)
Anaplastic carcinoma	2 (4.6)
Follicular carcinoma	2 (4.6)
Adenomatoid nodule with cystic change	1 (2.3)
Hyalinizing trabecular adenoma	1 (2.3)
MRI findings	
Malignant	8 (18.6)
Benign	35 (81.4)
Histopathological diagnosis	
Benign	35 (81.4)
Malignant	8 (18.6)

**Figure 1:**
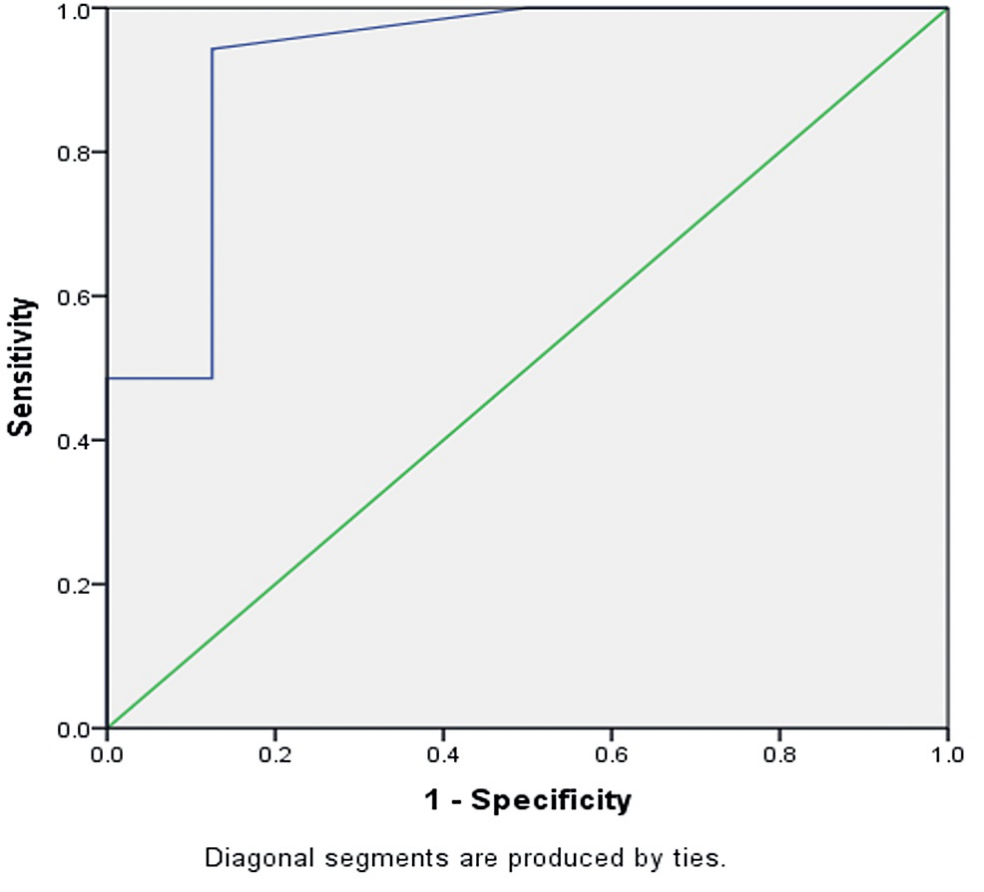
ROC analysis of predictive validity of ADC values of the thyroid nodules in predicting final HPE diagnosis of the thyroid nodules (N = 43; AUC = 0.925, p < 0.001). ROC, receiver operating characteristic; ADC, apparent diffusion coefficient; HPE, histopathological examination

As per histopathology findings, 35 (81.4%) nodules were benign and 8 (18.6%) nodules were malignant. According to ADC values on MRI, 8 (18.6%) nodules were malignant and 35 (81.4%) nodules were benign. The ADC value of thyroid nodules had excellent predictive validity in predicting malignancy, as indicated by the area under the curve of 0.925 (95% CI 0.804-1.000; *p *< 0.001; Figure 1).

ADC < 1 × 10^−3^ mm^2^/s had a sensitivity of 87.50% (95% CI 47.35%-99.68%) in predicting the final histopathology of the thyroid nodules, a specificity of 94.29% (95% CI 80.84%-99.30%), a false-positive rate of 5.71% (95% CI 0.70%-19.16%), and the total diagnostic accuracy of 93.02% (95% CI 80.94%-98.54%; Table 3).

**Table 3:** Predictive validity of ADC values of the thyroid nodules in predicting histopathological diagnosis (N = 43). ADC, apparent diffusion coefficient; CI, confidence interval

Parameter (ADC < 1)	Value	95% CI
Sensitivity	87.50%	47.35%-99.68%
Specificity	94.29%	80.84%-99.30%
False-positive rate	5.71%	0.70%-19.16%
False-negative rate	12.50%	0.32%-52.65%
Positive predictive value	77.78%	39.99%-97.19%
Negative predictive value	97.06%	84.67%-99.93%
Diagnostic accuracy	93.02%	80.94%-98.54%

## Discussion

This study assessed the role of DWI with ADC on MRI in differentiating benign from malignant thyroid nodules. DWI with ADC has a major role in differentiating benign from malignant thyroid nodules quantitatively by calculating ADC values. The ADC value of thyroid nodules has excellent predictive validity in differentiating benign and malignant nodules, as indicated by the area under the curve value of 0.925 (95% CI 0.804-1.000; *p *< 0.001). ADC values showed a sensitivity of 87.50% (95% CI 47.35%-99.68%), a specificity of 94.29% (95% CI 80.84%-99.30%), and a total diagnostic accuracy of 93.02% (95% CI 80.94%-98.54%).

According to these findings, DWI helps identify differences in cell density that can be used to discern benign from malignant thyroid nodules. Similarly, a study by Song et al. found that DWI and ADC measurements acted as a functional MRI modality for differentiating benign from malignant thyroid nodules [12]. In this study, the mean value of ADC was 1.42 ± 0.45 × 10^−3^ mm^2^/s (range: 0.51 to 2.90 × 10^−3^ mm^2^/s). Wang et al. reported that using the ADC value with the highest *b* value (2,000 s/mm^2^) to differentiate benign from malignant thyroid tumors yielded a sensitivity of 96.15% and a specificity of 85.48% [13]. Razek et al. used advanced diffusion imaging modules of the head and neck as a reference for future thyroid research [14].

The ADC value of thyroid nodules in this study has excellent predictive validity in predicting malignancy, as indicated by the area under the curve close to 0.925 (95% CI 0.804-1.000; *p *< 0.001). Linh et al. found that the area under the curve (ROC) of the test using ADC values to diagnose malignant thyroid nodules was very close to 1 (90% to 94%) [15]. In this study, the ADC cutoff value was <1 × 10^−3^ mm^2^/s, the sensitivity was 87.50%, the specificity was 94.29%, and the total diagnostic accuracy was 93.02%. Nakahira et al. found the cutoff point of ADC as 1.60 × 10^−3^ mm^2^/s and the sensitivity, specificity, and accuracy were 94.73%, 82.60%, and 88.09%, respectively, indicating these quantitative ADC values might be helpful for this differentiation [16].

From a clinical perspective, a pilot study conducted by Lu et al. found that DWI with ADC has clinical relevance in providing additional risk stratification for small thyroid tumors that are difficult to diagnose using ultrasound and clinical evaluation [17]. These observations can further be validated in larger prospective trials among patients being considered for a palliative approach rather than surgical resection where using DWI with ADC values on MRI could augment the standard preoperative ultrasound evaluation and avoid unnecessary fine needle aspiration cytology (FNAC) evaluation of the nodules [17].

DWI provides information about the cellularity and cell membrane architecture of tumoral tissue and can help in differentiating malignant and benign tumors. ADC values are usually overlapped in benign and malignant tumors. DWI provides information on tumors' response to treatment. Conventional MRI, DWI, and ADC values can be used in combination for additional information on patients. The absence of radiation, lack of intravenous contrast material, faster technique, and quantitative information of tissue as provided by ADC measurement are some of the advantages of DWI [18].

This study had a few limitations. The sample size was small, as the study was confined to a single center, and the findings cannot be generalized to a larger community. We do not have follow-up data from subjects that could have influenced our results. Further longitudinal multicentric studies with a large sample that includes various diseases are recommended to support this study's findings.

## Conclusions

We conducted this study to determine the role of DWI in differentiating benign from malignant thyroid nodules using ADC values and histopathology evaluations of thyroid nodules. DWI with ADC had excellent reproducibility and repeatability for differentiating benign from malignant thyroid nodules. Radiologists can use DWI routinely in oncologic settings for information on tumoral tissue. ADC can also be added as an adjunct to conventional MRI sequences.
